# An Improved WiFi Indoor Positioning Algorithm by Weighted Fusion

**DOI:** 10.3390/s150921824

**Published:** 2015-08-31

**Authors:** Rui Ma, Qiang Guo, Changzhen Hu, Jingfeng Xue

**Affiliations:** School of Software, Beijing Institute of Technology, Haidian District, Beijing 100081, China; E-Mails: guoqiangbit@gmail.com (Q.G.); chzhoo@bit.edu.cn (C.H.); xuejf@bit.edu.cn (J.X.)

**Keywords:** WiFi indoor positioning, location fingerprinting algorithm, weighted fusion

## Abstract

The rapid development of mobile Internet has offered the opportunity for WiFi indoor positioning to come under the spotlight due to its low cost. However, nowadays the accuracy of WiFi indoor positioning cannot meet the demands of practical applications. To solve this problem, this paper proposes an improved WiFi indoor positioning algorithm by weighted fusion. The proposed algorithm is based on traditional location fingerprinting algorithms and consists of two stages: the offline acquisition and the online positioning. The offline acquisition process selects optimal parameters to complete the signal acquisition, and it forms a database of fingerprints by error classification and handling. To further improve the accuracy of positioning, the online positioning process first uses a pre-match method to select the candidate fingerprints to shorten the positioning time. After that, it uses the improved Euclidean distance and the improved joint probability to calculate two intermediate results, and further calculates the final result from these two intermediate results by weighted fusion. The improved Euclidean distance introduces the standard deviation of WiFi signal strength to smooth the WiFi signal fluctuation and the improved joint probability introduces the logarithmic calculation to reduce the difference between probability values. Comparing the proposed algorithm, the Euclidean distance based WKNN algorithm and the joint probability algorithm, the experimental results indicate that the proposed algorithm has higher positioning accuracy.

## 1. Introduction

With the extensive development of mobile Internet spurred by the widespread usage of mobile devices and mobile communication technology, the demands on Indoor Positioning Service (IPS) increases unceasingly. As a result, many kinds of IPS apps have emerged in an endless stream [1,2,3]. The key of IPS is the positioning technology. Furthermore, the accuracy of a technology determines its long-term prospects.

Nowadays, outdoor positioning technology has matured. Global Positioning System [4] (GPS) has been widely used in outdoor environments and is well positioned. Since the GPS technology mainly relies on signal propagation in the air, buildings and their complex architecture will interfere with signal propagation, and therefore limit the usage of GPS in indoor environments. To meet the needs of indoor positioning, many researchers have found ways of using the existing indoor wireless communication technology, such as UWB, RFID, Bluetooth, Zig bee, WiFi. Because of the early development of WiFi networks, WiFi access points can be seen everywhere in indoor environments and almost all mobile devices have a built-in WiFi receiving module. As a result, WiFi indoor positioning has become an attractive research topic in developing indoor positioning.

When it comes to the common WiFi indoor positioning algorithm, the location fingerprinting algorithm has gained increasing attention as it does not require the location of WiFi access points. Yet, the fingerprinting approach suffers from two major problems in practical applications. On one hand, the site survey takes too much time and manpower during the offline acquisition process. On the other, the accuracy of the fingerprinting approach is far from adequate. Up to this point, many new researches have been put forward to address the problem of the time and energy costs, and they have worked out really well. However, for the accuracy, there is still a long way to go considering that most of the current researches cannot obtain accuracy within 2 m for practical application. Although some researches may get good accuracy within 2 m, their algorithms take too much time and calculation [5].

Therefore, to improve positioning accuracy of traditional location fingerprinting algorithm, this paper proposes an improved WiFi indoor positioning algorithm by weighted fusion. The proposed algorithm based on traditional location fingerprinting algorithm consists of two stages: Offline acquisition process and online positioning process. The offline acquisition process selects optimal parameters to complete the signal acquisition, and it forms a database of fingerprints by error classification and handling. The online positioning process first uses pre-match method to select the candidate fingerprints to shorten the time for positioning; then it uses the improved Euclidean distance and the improved joint probability to calculate two intermediate results; and it finally obtains the final result from these intermediate results by weighted fusion. The improved Euclidean distance using standard deviation is based on traditional Euclidean distance improved by the standard deviation of WiFi signal, which could consider the degree of WiFi fluctuation. The improved joint probability using logarithmic calculation is based on traditional joint probability improved by logarithmic calculation, which could consider the large difference of probability value. The final result has the advantages of both methods to improve the positioning accuracy. Through comparing the performance of the proposed algorithm, the Euclidean distance algorithm-WKNN algorithm and the joint probability algorithm, this paper verifies that the proposed algorithm has higher positioning accuracy.

The rest of the paper is organized as follows. In Section 2, this paper reviews related works. Section 3 provides the proposed algorithm. The experimental results and performance evaluation of the proposed algorithm are presented in Section 4. Section 5 concludes the future works.

## 2. Related Works

Traditional WiFi indoor positioning algorithm can be divided into three categories: Proximity algorithm, triangulation algorithm and scene analysis algorithm.

### 2.1. Proximity Algorithm

The proximity algorithm [6] helps to estimate the location of the target place using the proximity relationship between the target place and WiFi access points. When the mobile device at the target place receives WiFi signals from different WiFi access points, the location of WiFi access point with the strongest signal will be regarded as the location of the target place. The accuracy of this algorithm is determined by the distribution density and signal range of WiFi access points.

### 2.2. Triangulation Algorithm

The triangulation algorithm [7] helps to estimate the location of the target place based on geometric properties of triangles. When the mobile device at the target place receives the WiFi signals from one or more WiFi access points, the time of arrival (TOA), the angle of arrival (AOA) and the received signal strength (RSS) of WiFi signals will be used to calculate the distances between the target place and WiFi access points. With the locations of three or more WiFi access points, the target place can be estimated by triangulation.

### 2.3. Scene Analysis Algorithm

The scene analysis algorithm [8] refers to the type of algorithm that first collects features (fingerprints) of a scene and then estimates the target place of an object by matching online measurements with the closest *a priori* location fingerprints. RSS-based location fingerprinting algorithm is commonly used in scene analysis. Also magnetic field and even the value of GPS signal indoors (its level) has been used as fingerprints [9,10].

Traditional fingerprinting approach can be divided into two stages: offline stage and online stage. During the offline stage, a site survey is performed in an environment. The location coordinates and respective signal strengths from nearby WiFi access points are collected. During the online stage, a location positioning technique uses the currently observed signal strengths and previously collected information to figure out an estimated location. The main challenge to the techniques based on location fingerprints is that the received signal strength could be affected by diffraction, reflection, scattering and absorption during the propagation in indoor environments.

### 2.4. Related Researches

The proximity algorithm is simple but not as accurate. It is generally used to support outdoor positioning. The triangulation algorithm requires the location of WiFi access points, thus limits the range of application. The scene analysis algorithm has the advantage of accuracy and it does not require the location of WiFi access points, and thus plays an increasingly important role in the indoor positioning field.

The previous researches on offline stage are mainly focused on improving the quality of the database of fingerprints. For example, Husen [9] proposed that personal direction should be taken into consideration when collecting WiFi signal strengths. Galván-Tejada [10] presented an extension and improvement of current indoor localization model based on the feature extraction of 46 magnetic field signal features. By adding more auxiliary features to WiFi signal fingerprints in the offline stage, it will help the online positioning to be more accurate as in [9,10]. The management and utilization on the database of fingerprints were studied in [11,12,13,14]. Atia and Yoon [11,12] give their way to update the database of fingerprints automatically. Koweerawong and Jung [13,14] proposed two methods about how to organize the database of fingerprints. Although they did not improve the positioning accuracy, they managed to improve the efficiency of algorithms. In addition, Aomumpai [15] studied how to optimize the placement of collecting points, which could improve the location performance. As for the online stage, the previous researches on online stage mainly focused on how to improve the accuracy of positioning. Sánchez-Rodríguez and Chen [5,16] mentioned the sensor fusion, but extra sensors are necessary, which would increase the cost of positioning. Song [17] proposed a weighted fingerprinting approach based on the relationship between the average value and the standard deviation of WiFi signal strength. The data fusion method is applied to the WiFi positioning in [18]. Yang and Liu [19,20] both proposed new algorithms integrating traditional algorithms. At the same time, more and more researches adopted machine learning to propose new algorithms [21]. The researches on online stage are abundant, but some of them may take too much time and calculation. Whereas specific to positioning applications, Laoudias [22,23] presented indoor positioning systems developed for Android smartphones. However their accuracy is not adequate. That indicates that choosing between the precision and the practical application is worth considering.

Although the above researches have made some breakthroughs, there are still some downsides:

(1) The lack of researches on WiFi signal features.
(2) Deficiency in the comprehensiveness in the offline stage discounts error of collection.
(3) Too much time and calculation during the online stage.

## 3. An Improved WiFi Indoor Positioning Algorithm

**Figure 1:**
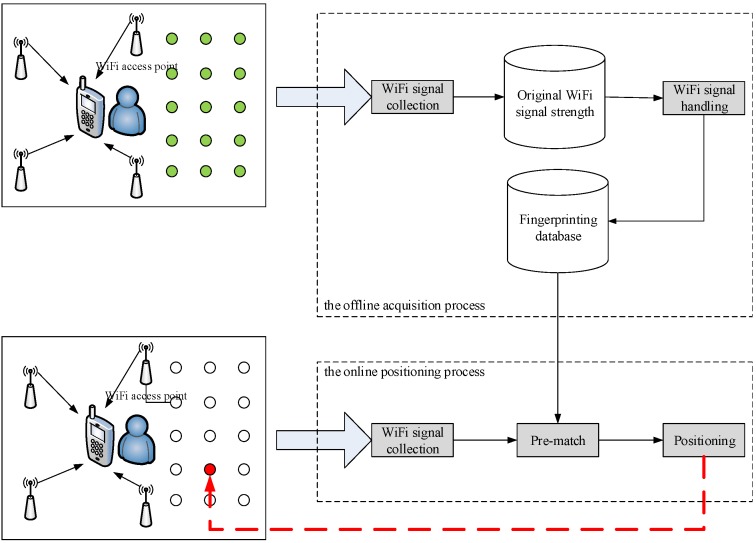
Methodology of proposed algorithm.

In this section, an extension and improved algorithm is presented for estimating the location of the target place. The proposed algorithm based on traditional location fingerprinting algorithm improves each step of traditional algorithm and uses the weighted fusion. It is shown in Figure 1.

### 3.1. Overview

The proposed algorithm is based on the traditional fingerprinting algorithm [8] and also consists of two stages: the offline acquisition process and the online positioning process. However, the proposed algorithm managed to be more precise.

The offline acquisition process consists of three phases:

Phase 1: Collecting Indoor WiFi signal

This phase collects the WiFi signal based on a map of collecting points. The map of collecting points is formed by dividing the positioning home into a grid of equidistant points. Then, the original WiFi signal is collected by using a mobile device at the location of every single point.

Phase 2: Error handling of Indoor WiFi signal collecting

This phase processes the original WiFi signal by classifying the error when collecting. There are three types of errors: systematic error, gross error, and random error.

Phase 3: Constructing the database of location fingerprints

The database of location fingerprints mainly contains the following information: the average value of original WiFi signal strength, the standard deviation of original WiFi signal strength, and the average value of processed WiFi signal strength.

The online positioning process consists of four phases:

Phase 1: Pre-matching location fingerprints

This phase is used to reduce the number of possible fingerprints, so as to shorten the time for positioning.

Phase 2: Improved Euclidean distance positioning

After pre-match, the improved Euclidean distance is used to get an intermediate positioning result (X_1_, Y_1_). As for the improved traditional Euclidean distance positioning, this work chooses possible K_d_ fingerprints and uses their location to estimate a result (X_1_, Y_1_). The improved Euclidean distance values of these fingerprints are the smallest ones in all possible fingerprints.

Phase 3: Improved joint probability positioning

After phase 2, the improved joint probability is used to get an intermediate positioning result (X_2_, Y_2_). In the improved joint probability positioning, this work chooses the possible K_p_ fingerprints and uses their location to estimate a result (X_2_, Y_2_). The improved joint probability values of these fingerprints are the largest ones in all possible fingerprints.

Phase 4: Weighted fusion positioning

This phase uses (X_1_, Y_1_) and (X_2_, Y_2_) to calculate the final result $\text{(}\bar{X}\text{, }\bar{Y}\text{)}$ by the weighted fusion method.

### 3.2. The Offline Acquisition Process

#### 3.2.1. Collecting Indoor WiFi Signal

Compared to real indoor positioning environment, this work uses 2-D modeling of the indoor structure without considering different floors. After modeling the positioning environment, this work divides the indoor positioning home into a grid of equidistant points. So, the location of each collecting point can be expressed as (ID, X, Y). ID stands for the identification of each collecting point, (X, Y) represents the coordinate of each collecting point. Then the original WiFi signal is collected by using a mobile device at the location of every collecting point. These original WiFi signals and their location information will be uploaded to the server.

#### 3.2.2. Error Handling of Indoor WiFi Signal Collecting

Since the complex indoor structure will lead to serious WiFi signal interference, the proposed algorithm deals with the original WiFi signal by classifying the collecting errors. There are three steps in the error handling:

Step 1: Systematic error handling

The systematic error is caused by the difference of hardware device. To reduce the influence on positioning accuracy, this work handles the systematic error by setting the offset value. The offset value is the difference between different hardware devices. It could be obtained as follows: We first set a two-dimensional code at the same place, and then use different devices to collect WiFi signals. The difference between different signals is defined as the offset value.

Step 2: Gross error handling

The gross error is caused by some burst interference like movement of people. To reduce the influence on positioning accuracy, this work handles the gross error by T test which is a common statistical method.

Step 3: Random error handling

Compared with the gross error, the random error is caused by the WiFi signal interference. The value of random error is smaller and could not be eliminated. Because the WiFi signal follows normal distribution basically, this work would use median filter to deal with the random error.

Special explanation is needed that because some access points have weak signal strength in indoor scenario, those signals sometimes may be missed. When the signal is missing, this work supposes that the received signal strength equals to −100 dbm as you can see in the following experiment.

#### 3.2.3. Constructing the Database of Location Fingerprints

The database of location fingerprints is formed by many location fingerprints. A location fingerprint can be expressed as (ID, X, Y, MAC_k_, AVG_k_, PAVG_k_, DEV_k_) where *k* = 1, 2, …, *n*. A location fingerprint stands for a collecting point. A collecting point could receive *n* WiFi signals, so a location fingerprint will contain *n* WiFi signal strengths. (ID, X, Y) represents the location of one collecting point. MAC_k_ stands for the physical address of the *k*th WiFi access point. AVG_k_ means the average value of the *k*th original WiFi signal strength. PAVG_k_ means the average value of the *k*th processed WiFi signal strength. DEV_k_ means the standard deviation of the *k*th original WiFi signal strength.

### 3.3. The Online Positioning Process

#### 3.3.1. Pre-matching Location Fingerprints

When the proposed algorithm is applied in the online positioning process, the WiFi signal information of the target place should be obtained at first. The WiFi signal information of the target place can be expressed as (MAC_k_, AVG_k_, PAVG_k_, DEV_k_) where *k* = 1, 2, …, *m*, which have the same meaning with the previous symbols (described in Section 3.2.3) but they are received at the target place. After that, the following contents will use the WiFi signal information to estimate the location of the target place. The pre-match method aims at reducing the number of possible location fingerprints and has two steps:

Step 1: From the WiFi signal information of the target place, MAC addresses will be found out if PAVG > FLAG, where FLAG is a value that is used to distinguish the stronger WiFi signal and FLAG equals to the average value of the total *k* PAVGs.

Step 2: The location fingerprints that contain above MAC addresses will be the possible fingerprints.

#### 3.3.2. Improved Euclidean Distance Positioning

Suppose that we have received *n* WiFi signals from different access points of the target place, and have got *m* possible fingerprints after pre-match. The improved Euclidean distance could be defined as:

(1) $$d_{i}=\sqrt{\sum_{k=1}^{n}{\left(|PAVG_{k}-PAVG_{ik}|+DEV_{k}+DEV_{ik}\right)}^{2}}$$

where *i* = 1,2, …, *m*, *k* = 1,2, …, *n*. *d*_i_ is the improved Euclidean distance value between the target place and the *i*th possible fingerprint. DEV_k_ is the standard deviation of the *k*th original WiFi signal strength received at the target place. DEV_ik_ is the standard deviation of the *k*th original WiFi signal strength in the *i*th possible fingerprint. PAVG_k_ is the average value of the *k*th processed WiFi signal strength received at the target place. PAVG_ik_ is the average value of the *k*th processed WiFi signal strength in the *i*th possible fingerprints. The processed WiFi signal comes from the original WiFi signal with error handling.

Then, this work will choose the possible K_d_ fingerprints, the smallest improved Euclidean distance values, and then use their locations to estimate (X_1_, Y_1_).

(2) $$(X_{1},Y_{1})=\frac{\sum_{j=1}^{K_{d}}\left[{\text{ω}}_{j}\times (X_{j},Y_{j})\right]}{\sum_{j=1}^{K_{d}}{\text{ω}}_{j}}$$

where ${\text{ω}}_{j}$ = $1/d_{j}$, (X_j_, Y_j_) is the location of the *j*th possible fingerprint. Compared with traditional Euclidean distance, the improved Euclidean distance introduces the standard deviation of WiFi signal so as to could take the WiFi fluctuation into consideration. Here the standard deviation of WiFi signal could reflect the degree of WiFi fluctuation.

The improved Euclidean distance using the standard deviation of WiFi signal strength could be better than the traditional Euclidean distance, the specific explanation would be provided in Section 4.4.

#### 3.3.3. Improved Joint Probability Positioning

Suppose that we have received *n* WiFi signals from different access points of the target place, and have got *m* possible fingerprints after pre-match. The improved joint probability could be defined as:

(3) $$P_{i}=P_{i1}\times P_{i2}\times \ldots \times P_{ik}\times \ldots \times P_{in}$$

where *i* = 1,2, …, *m*, *k* = 1,2, …, *n*, P_i_ is the joint probability value between the target place and the *i*th possible fingerprint, P_ik_ is the joint probability of the *k*th original WiFi signal strength in the *i*th possible fingerprint.

(4) $$P_{ik}=\frac{1}{\text{σ}\sqrt{2\text{π}}}e^{-\frac{{\left(x-\text{μ}\right)}^{2}}{2{\text{σ}}^{2}}}$$

where $\text{μ}=AVG_{ik}$, $\text{σ}=DEV_{ik}$, $x=AVG_{k}$, AVG_k_ is the average value of the *k*th original WiFi signal strength received at the target place, AVG_ik_ is the average value of the *k*th original WiFi signal strength in the *i*th possible fingerprint, DEV_ik_ is the standard deviation of the *k*th original WiFi signal strength in the *i*th possible fingerprint.

After that, this work will choose the possible K_p_ fingerprints of the largest improved joint probability values, and then use their locations to estimate (X_2_, Y_2_).

(5) $$(X_{2},Y_{2})=\frac{\sum_{j=1}^{K_{p}}\left[{\text{ω}}_{j}\times (X_{j},Y_{j})\right]}{\sum_{j=1}^{K_{p}}{\text{ω}}_{j}}$$

where ${\text{ω}}_{j}$ = ${\mathrm{lgP}}_{j}$, (X_j_, Y_j_) is the location of the *j*th possible fingerprint. In most of previous researches, ${\text{ω}}_{j}$ = $P_{j}$ is common but there is a problem that although the value of every P_j_ is small, the order of magnitude of all values is very different. If we just use P_j_ as ${\text{ω}}_{j}$, the Equation (5) might make no sense, and the result (X_2_, Y_2_) would coincide with the location of the largest P_j_. Therefore, to make ${\text{ω}}_{j}$ sensitive, this work uses logarithmic calculation to process P_j_ to get a more accurate result of (X_2_, Y_2_).

#### 3.3.4. Weighted Fusion Positioning

Using weighted fusion, this work calculates the variance D_1_ of K_d_ shortest Euclidean distance as well as the variance D_2_ of K_p_ largest joint probability. Then the final result $\text{(}\bar{X}\text{, }\bar{Y}\text{)}$ can be expressed as:

(6) $$\left(\bar{X},\bar{Y})=\frac{D_{1}}{D_{1}+D_{2}}\times (X_{1},Y_{1})+\frac{D_{2}}{D_{1}+D_{2}}\times (X_{2},Y_{2}\right)$$

The value of the variance reflects the accuracy of the intermediate result. That is to say, one of the corresponding K fingerprints is very similar to the target place. It could be concluded that the larger one is relatively important in estimating the location of the target place and could be assignment a higher weight. Based on this point, the final result has the advantages of both methods so that it can improve the positioning accuracy.

## 4. Simulation Results and Evaluation

### 4.1. Simulation Environment

**Figure 2:**
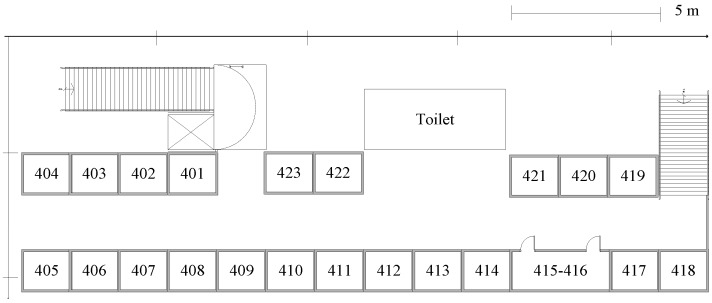
The fourth floor of software building layout.

**Figure 3:**
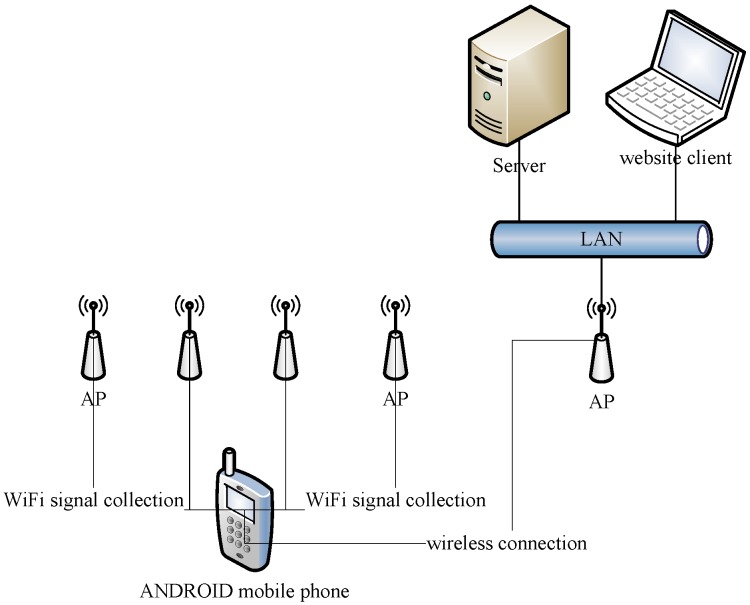
Prototype system framework.

This work collects data in a typical indoor scenario as shown in Figure 2. The simulation experiment is done on the fourth floor of the software building at the Beijing Institute of Technology. The prototype system frame is shown in Figure 3.

The prototype system contains three parts: the server, the mobile client, and the website client. The server is responsible for positioning calculation, the mobile client is responsible for collecting WiFi signals and passing the data to the server, and the website client is responsible for displaying the result by graph. The server has CPU: Intel(R) Core(TM) i5 CPU M430 @2.27 GHz and 2.00 GB RAM. The mobile device is Huawei Honor 3C.

### 4.2. Indoor WiFi Signal Collection

**Figure 4:**
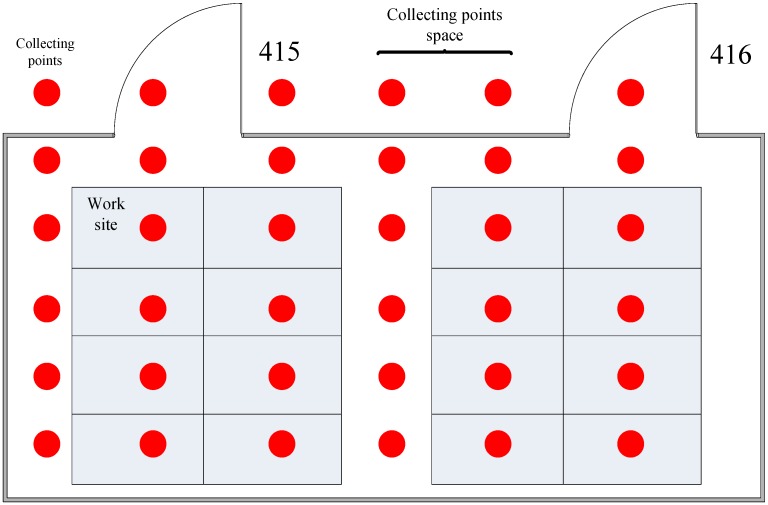
Distribution of collecting points.

The test bed is in room 415–416 at the fourth floor of the software building. The room is of 5 m × 10 m, and Figure 4 shows the distribution of collecting points. The collecting parameters can be seen in Table 1.

**Table 1:** WiFi signal collecting parameters.

Parameters	Value	Comments
K_d_ and K_p_	4	coming from experiment result
collecting spacing	1 m	coming from paper [17]
collecting frequency	10 Hz	determined by the mobile device
collecting time	10 s	determined by actual demands
number of points	36	determined by room size

**Figure 5:**
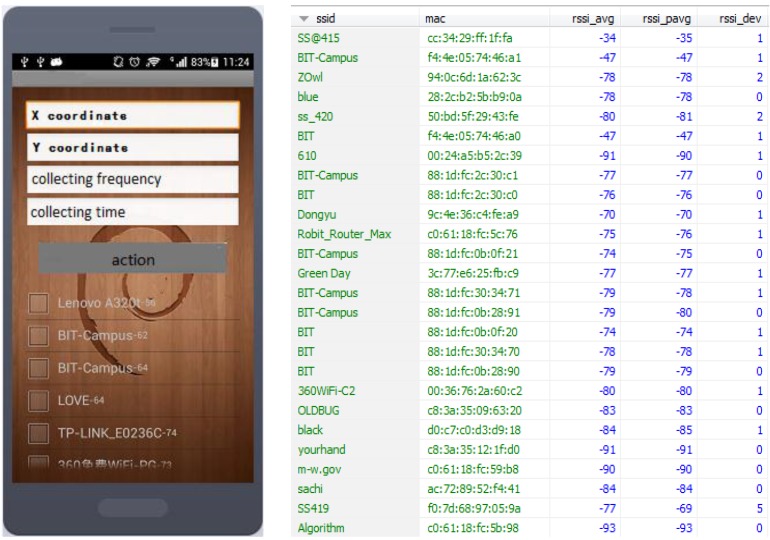
The mobile client and one fingerprint.

Figure 5 shows the mobile client and one fingerprint, where *ssid* and *mac* are the SSID and MAC address of WiFi access points, *rssi_avg* is the average value of the original WiFi signal strength, *rssi_pavg* is the average value of processed WiFi signal strength, *rssi_dev* is the standard deviation of the original WiFi signal strength.

### 4.3. Indoor WiFi Signal Error Handling

**Figure 6:**
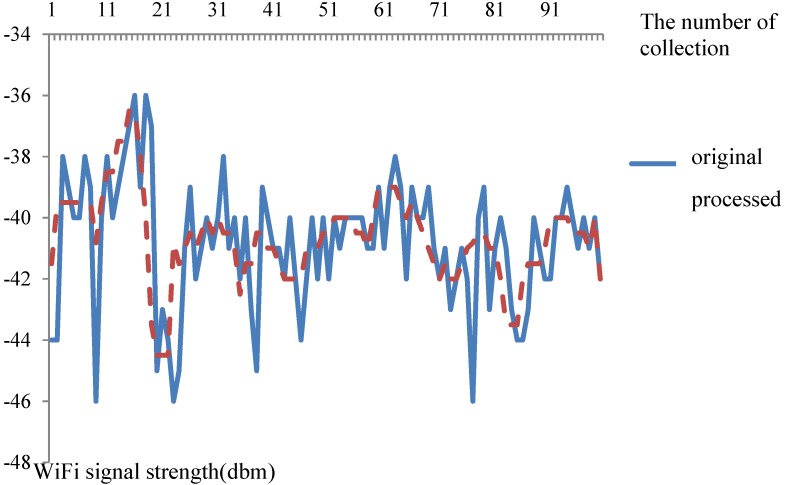
WiFi signal error handling.

To verify that error handling will reduce signal fluctuations, this work compares waveforms of the original and the processed WiFi signal, as is shown in Figure 6.

The error handling contains: systematic error handling, gross error handling, and random error handling. It can be seen from Figure 6 that the signal fluctuation becomes smoother after error handling. The original signal demonstrates great strength fluctuations compared with the processed signal.

To verify that the Euclidean distance calculated by processed WiFi signal could improve the accuracy of positioning, this work calculates the Euclidean distance of two collections at the same position, as is shown in Table 2. In this table, *mac* stands for the MAC address of WiFi access points, *rssi_avg1* and *rssi_pavg1* are the average value of the original and the processed WiFi signal for the first time, *rssi_avg2* and *rssi_pavg2* are the average value of the original and processed WiFi signal for the second time. As we can see in Table 2, −100 dbm means some WiFi signals are missed.

Table 2 illustrates that the Euclidean distance value calculated by the processed WiFi signal is smaller than the Euclidean distance value calculated by the original WiFi signal. It suggests that the Euclidean distance calculated by the processed WiFi signal is better.

In this example, the Euclidean distance of these two collections have only a little different. The reason is there are only a few people in the experimental environment. If the experimental environment becomes signal-complex, the difference will become more obvious.

**Table 2:** Euclidean distance of two collections at the same position.

MAC	Original		Processed	
	Rssi_avg1	Rssi_avg2	Rssi_pavg1	Rssi_pavg2
00:24:a5:b5:2c:39	−88	−86	−87	−86
3c:77:e6:25:fb:c9	−89	−86	−88	−86
50:bd:5f:29:43:fe	−81	−79	−81	−79
60:6c:66:1c:d1:81	−85	−85	−85	−85
88:1d:fc:0b:0f:20	−75	−73	−75	−73
88:1d:fc:0b:0f:21	−75	−75	−75	−74
88:1d:fc:0b:28:90	−75	−75	−75	−75
88:1d:fc:0b:28:91	−75	−75	−75	−75
88:1d:fc:2c:30:c0	−77	−75	−76	−76
88:1d:fc:2c:30:c1	−77	−75	−77	−76
88:1d:fc:30:34:70	−78	−78	−78	−79
88:1d:fc:30:34:71	−78	−78	−78	−78
94:0c:6d:1a:62:3c	−79	−79	−79	−79
9c:4e:36:c4:fe:a9	−75	−75	−74	−73
ac:72:89:52:f4:41	−86	−88	−86	−88
c0:61:18:fc:59:b8	−86	−85	−86	−85
c0:61:18:fc:5c:76	−78	−77	−78	−77
c8:3a:35:09:63:20	−84	−83	−84	−83
c8:3a:35:12:1f:d0	−91	−100	−91	−100
c8:3a:35:56:62:60	−90	−100	−90	−100
cc:34:29:ff:1f:fa	−39	−37	−38	−38
d0:c7:c0:d3:d9:18	−86	−87	−85	−87
ec:88:8f:65:49:a2	−90	−100	−90	−100
f0:7d:68:97:05:9a	−69	−69	−70	−69
Euclidean distance	17.94		17.61	

### 4.4. Features of Indoor WiFi Signal

According to the experimental analysis of WiFi signal, the features of the indoor WiFi signal could be concluded in three points:

1. The WiFi signal strength decreases with increasing distance.

This feature is caused by the WiFi signal decay during the transmission. The most commonly-used path loss model [24] for indoor environment is the International Telecommunication Union (ITU) indoor propagation model.

2. The WiFi signal follows normal distribution [25].

This feature is the fundamental theory of the improved joint probability positioning. A lot of researches have proved this feature both theoretically and experimentally [25,26]. Of course, there are some researchers who believe that the WiFi signal does not follow Gaussian distribution and is asymmetric, and further would like to use other models to describe the distribution of WiFi signal. However, our work still uses the Gaussian distribution. The reason is that the Gaussian distribution is relatively simple, but it really could describe the WiFi signal basically in our real scenario. Certainly, there must be some errors. Since the WiFi signal fluctuates inherently, the error is difficult to avoid.

3. The standard deviation of WiFi signal strength increases with the average value of WiFi signal strength.

**Figure 7:**
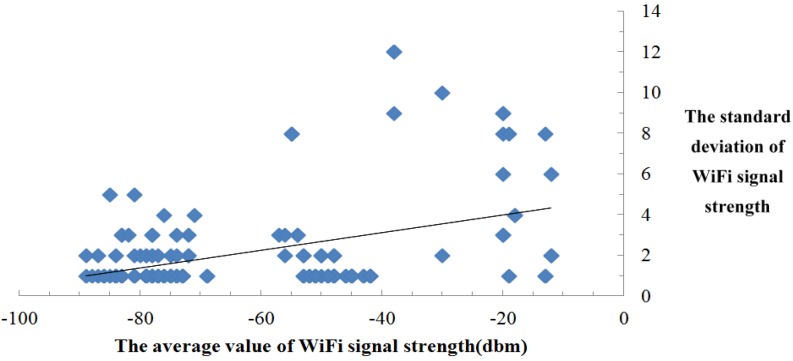
Feature of WiFi signal.

This feature is concluded from the experimental data, as shown in Figure 7. The main reason of obtaining this feature is that the closer the mobile device is to the WiFi hotspot, the greater the interference from the bodies of the signal collecting staff. This phenomenon directly results in larger standard deviation of WiFi signal.

Mathematically, the standard deviation of WiFi signal strength reflects the degree of WiFi signal fluctuation, so the third feature could also be described as: The degree of WiFi signal fluctuation increases with the average value of WiFi signal strength. It should be noted that the larger the WiFi signal fluctuation is, the greater the error in using this WiFi signal to estimate the location becomes. That phenomenon prompted us to consider merging the standard deviation of WiFi signal strength into the traditional Euclidean Distance.

As a result, this feature is applied to the improved Euclidean distance positioning in the proposed algorithm. The traditional Euclidean distance positioning is a way of comparing vector distance between the target place and the fingerprints. The smaller the Euclidean distance is, the closer the fingerprint is to the target place. Therefore, the traditional Euclidean distance does not consider the degree of WiFi signal fluctuation. To solve the problem, this work introduces the standard deviation of WiFi signal strength to the traditional Euclidean distance, as is shown in Equation (1). The improved Euclidean Distance could more accurately reflect the degree of similarity between the fingerprints and the target place.

### 4.5. Comparison with Other Positioning Algorithms

To verify the validity of the proposed algorithm, this work compares traditional WKNN algorithm, traditional joint probability algorithm and the proposed algorithm. It is defined that the location estimation error [27] *Dis_err* is the distance between the real location coordinates (*x*_0_, *y*_0_) and the system estimated location coordinates (*x*, *y*):

(7) $$Dis\_err=\sqrt{{\left(x-x_{0}\right)}^{2}+{\left(y-y_{0}\right)}^{2}}$$

#### 4.5.1. The Average Error of 100 Positioning

**Figure 8:**
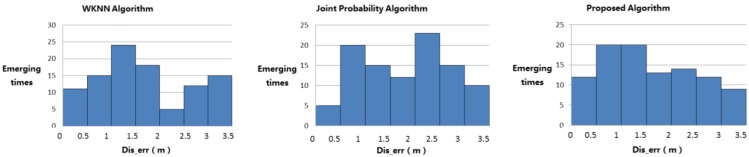
Distribution of the error of 100 positioning.

Figure 8 shows the distribution of the error of 100 positioning.

By calculating the average error of 100 positioning, the results are listed as follows:

(1) The traditional WKNN algorithm is 1.66 m.
(2) The WKNN algorithm based on improved Euclidean distance is 1.60 m.
(3) The traditional joint probability algorithm is 1.93 m.
(4) The joint probability algorithm based on improved joint probability is 1.87 m.
(5) The proposed algorithm is 1.54 m.

By comparing results (1) and (2), it is concluded that the improved Euclidean distance could improve the accuracy of traditional WKNN algorithm. By comparing results (3) and (4), it is found that the improved joint probability could improve the accuracy of traditional joint probability algorithm. By comparing results (1), (3) and (5), we find that the proposed algorithm has a better accuracy than those two traditional algorithms.

#### 4.5.2. The Probability Distribution of 100 Positioning

**Figure 9:**
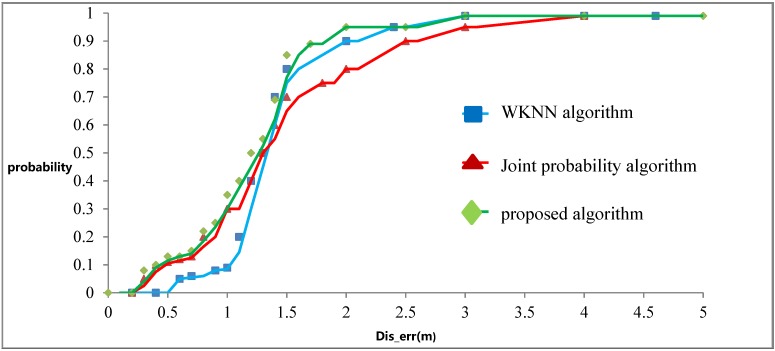
Probability distribution of 100 positioning.

Figure 9 shows that the probability of the three algorithms within different *Dis_err*. For example, as for the WKNN algorithm, if the *Dis_err* is within 1 m, the probability of it is about 0.09.

The data in Figure 9 leads to the following conclusions:

(1) In WKNN algorithm, 90% of Dis_err is less than 2 m; in joint probability algorithm, 70% of Dis_err is less than 2 m. So the WKNN algorithm is more accurate than the joint probability algorithm.
(2) The error based on the WKNN algorithm is about 1.5 m.
(3) The error based on the joint probability algorithm is of 1–2 m.
(4) The proposed algorithm combines the advantages of these two traditional algorithms. If the accuracy of WKNN algorithm is higher, the proposed algorithm will approach it; if the accuracy of joint probability algorithm is higher, the proposed algorithm will approach that. As a result, the accuracy of proposed algorithm is higher.

To sum up, the reasons why the proposed algorithm has higher accuracy are the following:

(1) The improved Euclidean distance could improve the accuracy of the traditional WKNN algorithm.
(2) The improved joint probability could improve the accuracy of the traditional joint probability algorithm.
(3) The proposed algorithm could combine both advantages of the two traditional algorithms and thus achieves better accuracy than the two traditional algorithms.

### 4.6. Impact of K Value

**Figure 10:**
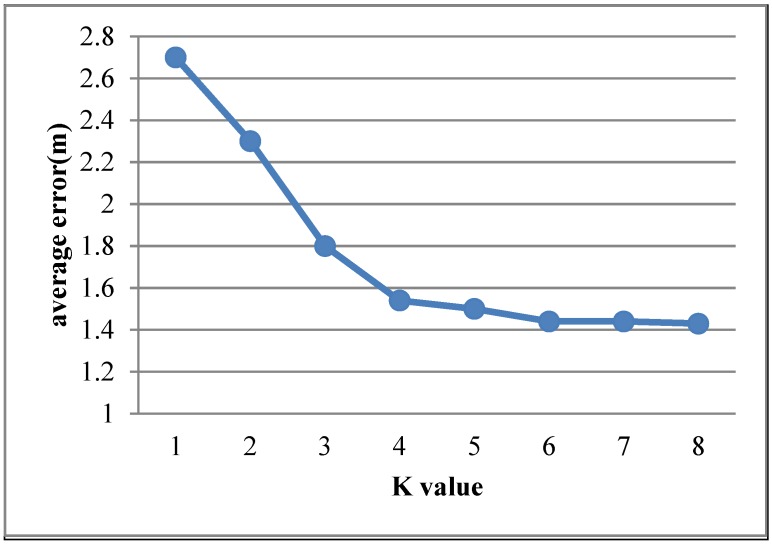
Average error of different K value.

In the proposed algorithm, the value of K_d_ and K_p_ influence the accuracy of the algorithm. In this work, we suppose that K_d_ and K_p_ are all equal to K. The experiment aims at finding the best *K* value for the proposed algorithm. Figure 10 shows that the average error of different *K* values.

Figure 10 shows that when *K* < 4, the average error will decrease with *K* value; when *K* ≥ 4, the average error remains stable. In this experiment, we choose the K_d_ and K_p_ at 4.

### 4.7. Impact of Collecting Point’s Spacing

**Figure 11:**
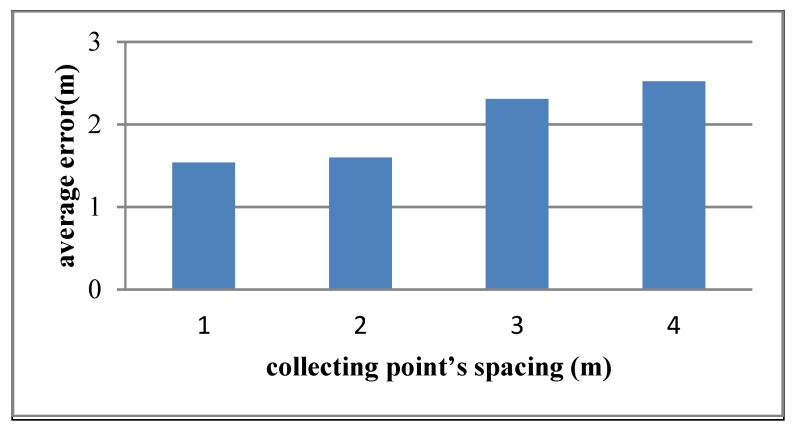
Average error of different collecting point’s spacing.

In the offline acquisition process of the proposed algorithm, the collecting point’s spacing will impact on the accuracy of the algorithm. Paper [26] suggests that the accuracy of algorithm will not be improved if collecting point’s spacing is too small. So this experiment selects 1 m, 2 m, 3 m, and 4 m as collecting point’s spacing during the offline acquisition process. The result is shown in Figure 11.

Figure 11 shows that when the collecting point’s spacing is 1 m or 2 m, the influence on the accuracy of proposed algorithm is weaker. However, if the collecting point’s spacing increases 3 m or 4 m, the average error becomes larger. So, the collecting point’s spacing is better set between 1 m and 2 m.

### 4.8. Impact of Human Body

**Figure 12:**
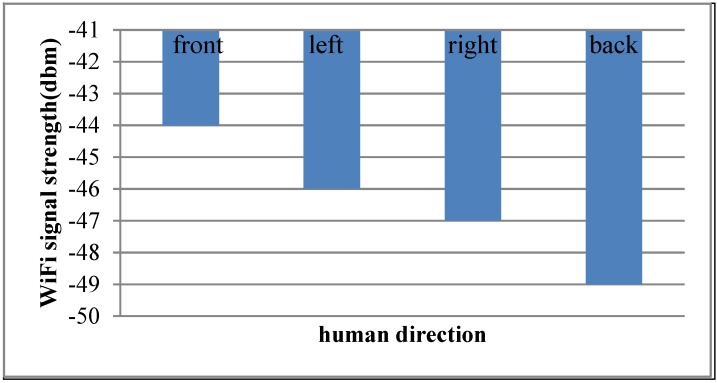
WiFi signal strength in different human movement directions.

The impacts of human body [9] are of two aspects: 1. The movement of humans when positioning; 2. The interference of human movement direction when collecting. The first aspect has been considered in the proposed algorithm. As for the second aspect, Figure 12 shows the WiFi signal strength in different human directions. In this experiment, we suppose that there is an AP only one meter away from the user.

It can be seen from Figure 12 that human movement direction has an effect on the accuracy. To mitigate this influence, this work assumes that heading-orientated human direction is always to the front.

## 5. Conclusions

WiFi indoor positioning depends on the WiFi wireless technology to obtain indoor location information, which is of great significance to the development of indoor positioning applications. Our work mainly focuses on the improvement of current traditional positioning algorithms and further proposes an improved WiFi indoor positioning algorithm by weighted fusion. The proposed algorithm is based on the traditional location fingerprinting algorithm. By using the WiFi signal error handling, better fingerprints during the offline acquisition process could be acquired. After improving the traditional Euclidean distance positioning and the joint probability positioning, a more accurate location result is achieved.

In the proposed algorithm, besides positioning accuracy, reducing labor costs is an important issue for researchers as well. It is also the future direction for this proposed work. To reduce labor costs, the future work can be focused on the following directions: 1. The way to construct the database of fingerprints automatically; 2. Once the database of fingerprints has been established, the problem of maintaining it without human interference should be solved.
